# The molecular signatures of compatible and incompatible pollination in Arabidopsis

**DOI:** 10.1186/s12864-021-07503-7

**Published:** 2021-04-14

**Authors:** Chie Kodera, Jérémy Just, Martine Da Rocha, Antoine Larrieu, Lucie Riglet, Jonathan Legrand, Frédérique Rozier, Thierry Gaude, Isabelle Fobis-Loisy

**Affiliations:** 1 Laboratoire Reproduction et Développement des Plantes, Univ Lyon, ENS de Lyon, UCB Lyon 1, CNRS, INRAE, Inria, F-69342 Lyon, France; 2 Present Address: Institut Jean-Pierre Bourgin, INRAE, AgroParisTech, Université Paris-Saclay, 78000 Versailles, France; 3 INRAE, Université Côte d’Azur, CNRS, ISA 400 route des Chappes BP 167, F-06903 Sophia Antipolis Cedex, France; 4 Present Address: Centre for Plant Sciences, Faculty of Biological Sciences, University of Leeds, Leeds, UK; 5 Present Address: Sainsbury Laboratory, Cambridge University, Cambridge, CB2 1LR UK

**Keywords:** RNA sequencing, SNPs, Pollen-stigma interaction, Compatible / incompatible pollination, Male-female transcriptome, PTI pathway

## Abstract

**Background:**

Fertilization in flowering plants depends on the early contact and acceptance of pollen grains by the receptive papilla cells of the stigma. Deciphering the specific transcriptomic response of both pollen and stigmatic cells during their interaction constitutes an important challenge to better our understanding of this cell recognition event.

**Results:**

Here we describe a transcriptomic analysis based on single nucleotide polymorphisms (SNPs) present in two *Arabidopsis thaliana* accessions, one used as female and the other as male. This strategy allowed us to distinguish 80% of transcripts according to their parental origins. We also developed a tool which predicts male/female specific expression for genes without SNP. We report an unanticipated transcriptional activity triggered in stigma upon incompatible pollination and show that following compatible interaction, components of the pattern-triggered immunity (PTI) pathway are induced on the female side.

**Conclusions:**

Our work unveils the molecular signatures of compatible and incompatible pollinations both at the male and female side. We provide invaluable resource and tools to identify potential new molecular players involved in pollen-stigma interaction.

**Supplementary Information:**

The online version contains supplementary material available at 10.1186/s12864-021-07503-7.

## Background

In flowering plants, the early interaction between the extremity of the female organ (stigma) and the male gametophyte (pollen grain) acts as a checkpoint for fertilization. This first step of the female-male interaction includes recognition by the female tissues of the male partner. In the Brassicaceae, sophisticated mechanisms have evolved that allow the epidermal cells of the stigma, also known as papillae, to reject self (or incompatible) pollen while accepting non-self (or compatible) pollen. These self/non-self recognition mechanisms underlie self-incompatibility and promote genetic variability in the species. Following compatible pollination, the dry pollen grain hydrates on the stigma papilla and ultimately germinates, producing a tube that penetrates the wall of the stigmatic cell and grows down to convey the male gametes towards the ovules for fertilization [1, 2]. By contrast, when a pollen grain is recognized as incompatible, it fails to hydrate properly and shows defective germination [3]. This rejection mechanism is initiated by a ligand-receptor interaction and is genetically controlled by a single polymorphic locus, called the *S*-locus [4]. The *S*-locus Cysteine Rich protein (SCR)/ *S*-locus protein 11 (SP11) located on the pollen surface interacts with its cognate *S*-locus Receptor Kinase (SRK) localized at the plasma membrane of the papilla cells [5, 6]. This interaction leads to the phosphorylation of SRK that triggers the downstream cascade leading to pollen rejection [1, 6, 7]. Cellular events triggered in the stigma papillae by these two pathways, compatible and incompatible, have started to be more clearly defined. Compatible pollination induces actin network orientation, calcium export and polarized secretion towards the pollen grain [8–11]. Incompatible pollen leads to inhibition of both actin polymerization and vesicular trafficking accompanied by a strong calcium influx within the stigmatic cell [9, 11, 12]. Stigmatic calcium fluxes were reported to involve the autoinhibited Ca^2+^-ATPase13 for pollen acceptance [8] and a glutamate receptor-like channel for pollen rejection [12]. In addition, the stigmatic EXO70A1 protein was identified as a factor required for polarized secretion during compatible pollination, which is negatively regulated in incompatible reaction [11, 13]. While these features are now well established, they constitute a narrow framework that limits the understanding of the whole recognition process. To obtain a global picture of the early fertilization events with no a priori, transcriptome approaches were conducted. The main goal was to draw up catalogues of genes whose expression is modulated during pollination so as to unravel the stigmatic response to compatible or incompatible pollen [8, 14–17]. However, the main drawback of these approaches was the impossibility to distinguish pollen and stigma derived transcripts. To address that issue, translatome analysis [18] has been applied to identify sex-specific genes expressed during pollination, but this strategy needed large amounts of tissues from a transgenic line expressing tagged ribosomes in pollen and fine techniques of biochemistry.

Here, inspired by a previous RNA-seq analysis [19], we developed a new experimental procedure, coupled with a bioinformatic analysis of sequencing data, to comprehensively unravel the dynamic events that occur both in the stigma and pollen grain following compatible and incompatible pollinations. We took advantage of the SNPs existing between two distinct *Arabidopsis thaliana* accessions, one used as female (Col-0) and the other as male (C24), to differentiate male and female transcripts based on a new statistical methodology, ASE-TIGAR [20]. This statistical tool can take all sequenced reads in account even those without SNPs, while previous study used only reads with SNPs [19]. Our analysis allowed the identification of 80% of mRNAs according to their parental origin and revealed transcriptional changes occurring specifically in either the stigma or pollen grain/tube. We report an unanticipated transcriptional activity triggered in stigma upon incompatible pollination. On the other hand, based on Gene Ontology enrichment study and pathway-based prediction of upregulated genes we show that following compatible pollination, components of the pattern-triggered immunity (PTI) pathway are induced on the female side. This work provides a key resource to identify potential new molecular players involved in pollen-stigma interaction.

## Results

### Experimental setup to isolate transcripts from compatible and incompatible pollinations in *A. thaliana*

**Fig. 1:**
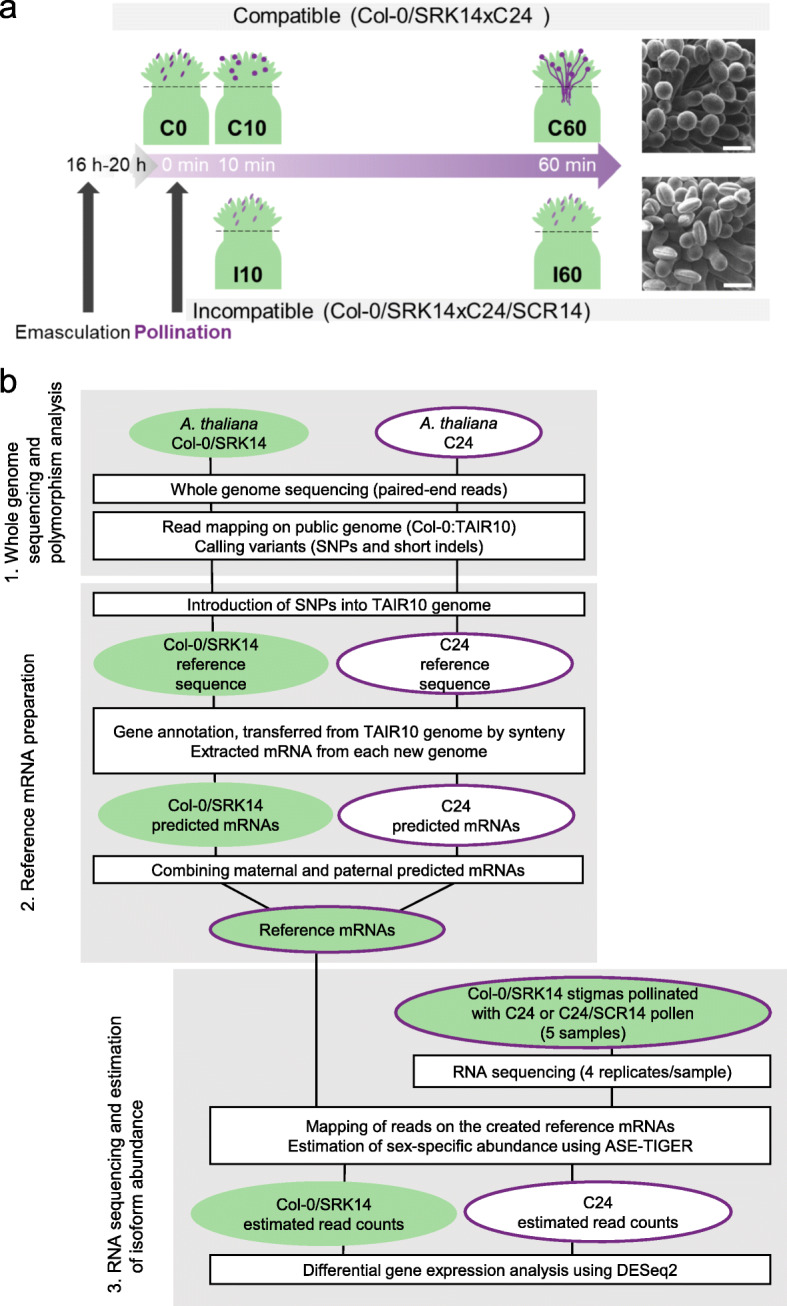
Experimental and data analysis pipeline of SNP-based RNA-seq analysis. **a** Time course of sample collection. Flowers of Col-0/*SRK14* were emasculated at stage 12, 16 h - 20 h before pollination with compatible (C24) or incompatible (C24/*SCR14*) pollen grains, respectively. Stigmas were harvested (dashed line) 0, 10, 60 min after compatible (C0, C10, C60) or incompatible (I10, I60) pollination for RNA extraction. Typical scanning electron microscope images observed 60 min after pollination, for compatible reaction (Col-0 /*SRK14* x C24) with hydrated round pollen grains and pollen tubes, and incompatible reaction (Col-0 /*SRK14* x C24/*SCR14*) with dehydrated ellipsoidal pollen grains and no pollen tube. Scale bar = 20 μm. **b** We performed whole genome sequencing and detected variants using GATK (McKenna et al., 2010). SNPs and short indels between Col-0/*SRK14* and C24 were identified (1.). Then, we produced new reference genomes for Col-0/*SRK14* and C24 introducing the identified SNPs into TAIR10 Col-0 genome (2.). After deriving predicted mRNA from the new genomes, we performed RNA-sequencing and got sex-specific isoform abundance by using a statistic tool ASE-TIGAR (Nariai et al., 2016) (3.). Read normalization and differentially expressed gene analysis were performed using DESeq2 (Love et al., 2014). Sequence quality was checked by FastQC (http://www.bioinformatics.babraham.ac.uk/projects/fastqc). DNA and RNA reads were cleaned with custom Perl scripts

Early work showed that self-incompatibility can be restored in the self-fertile species *A. thaliana* by reintroducing a functional *SRK-SCR* gene pair isolated from its close self-incompatible relative *A. lyrata* [21, 22]. To analyze both compatible and incompatible reactions and take advantage of nucleotide polymorphisms between *A. thaliana* accessions, previously, we generated two transgenic lines: one in the Col-0 background expressing the *SRK* gene from the *A. lyrata S*14 haplotype (Col-0/*SRK14*) and the other in C24 background expressing the *SCR* gene from the same *S*-haplotype (C24/*SCR14*) [3]. Both lines were self-fertile but when stage 13-14E (according to [23]) Col-0/*SRK14* stigmas were pollinated with C24/*SCR14* pollen, a strong incompatible reaction was observed as deduced from the absence of pollen tube in the stigma, 1 hour after pollen deposition (Fig. 1a).

Using these two Arabidopsis lines, we performed a compatible and incompatible pollination kinetics focusing on two time-points of the interaction to identify genes whose expression is modified following pollen-stigma interaction. We selected an early stage (10 min after pollen deposition), which corresponds to the start of pollen grain hydration [3], and a later stage (one hour after pollination) when pollen tubes reach the base of the stigma. We sequenced mRNAs extracted from pollinated stigmas at 10, and 60 min after compatible (Col-0/*SRK14* x C24) pollination (C10, C60, respectively) and incompatible (Col-0/*SRK14* x C24/*SCR14*) pollination (I10, I60, respectively) (Fig. 1a). We also harvested pollinated stigma right after compatible pollen deposition, as a control for both compatible and incompatible pollinations (C0).

### SNP-based transcriptome analysis using variants between Col-0 and C24

To distinguish the parental origin of the transcripts, we developed a method based on the detection of small genomic variations, including SNPs and small insertions and deletions (indels) between Col-0 and C24 accessions. The pipeline includes three main steps (Fig. 1b). In the first step, variations between Col-0/*SRK14* and C24 genomes were identified by whole-genome sequencing of the two strains with a read depth of 9.3 X for Col-0/*SRK14* and 14.2 X for C24 (Additional file 1: Table S1). After cleaning, the reads were mapped on the public Arabidopsis genome sequence retrieved from the Arabidopsis information resource (TAIR) database (TAIR10, accession Col-0). Single-nucleotide polymorphisms (SNPs) and short insertions/deletions (indels) were identified between the mapped reads and the TAIR10 sequence. Reads from Col0/*SRK14* and C24 covered 95.8 and 90.6% of the TAIR10 genome sequence, respectively. We identified 2032 variants (SNPs and indels) between Col-0/*SRK14* reads and TAIR10 genome sequence, while we identified 732,767 variants between C24 reads and TAIR10 genome sequence. In a second step, we generated two new genome sequences, one for Col-0/*SRK14* and one for C24, by introducing the SNPs identified in each line into the TAIR10 sequence. To simplify this step, only SNPs were used, and not indels (Fig. 1b). The two resulting genome sequences were compared by pairwise aligning their pseudomolecules, and we identified 616,781 SNPs between them. These two genome sequences were used as references for the subsequent steps of the project. We found that 27% of this polymorphism was in untranslated regions (UTRs) and coding sequences (CDSs) in Col-0/*SRK14* genome and 31% in C24 genome and led to sequence variations in predicted mRNAs. We then pairwise aligned the pseudomolecules of each of the two new genomes to their TAIR10 counterparts, and used both the position and the sequence information of TAIR10 gene models to annotate Col-0/SRK14 and C24 genome sequences. We predicted 39,205 gene models in Col-0/*SRK14* and 39,206 in C24. We extracted predicted mRNA sequences for each gene model from these annotated Col-0/*SRK14* and C24 genome sequences to produce maternal and paternal reference transcripts. Combining both sets of predicted mRNAs, we obtained a list of 39,204 predicted gene models that were shared between maternal and paternal references. The number of predicted mRNAs with at least one SNP between Col-0/*SRK14* and C24 was 31,271 among the total common predicted mRNAs (39204). This result allowed us to distinguish the origin of about 80% (31,271/39204 = 79.8%) of mRNAs with SNP-based analysis. The third step included mapping of the sequenced RNA reads and the estimation of sex-specific isoform abundance using ASE-TIGAR. The total length of raw reads from each condition was more than 6400 Mb (Additional file 1: Table S2). ASE-TIGAR uses a Bayesian approach to estimate allele-specific expression [20] and allowed us to utilize all sequenced reads even those without SNP to estimate gene expression. After obtaining the estimated read counts from ASE-TIGAR, we used DESeq2 [24] to normalize counts, for female and male transcripts, respectively (Fig. 1b).

**Fig. 2:**
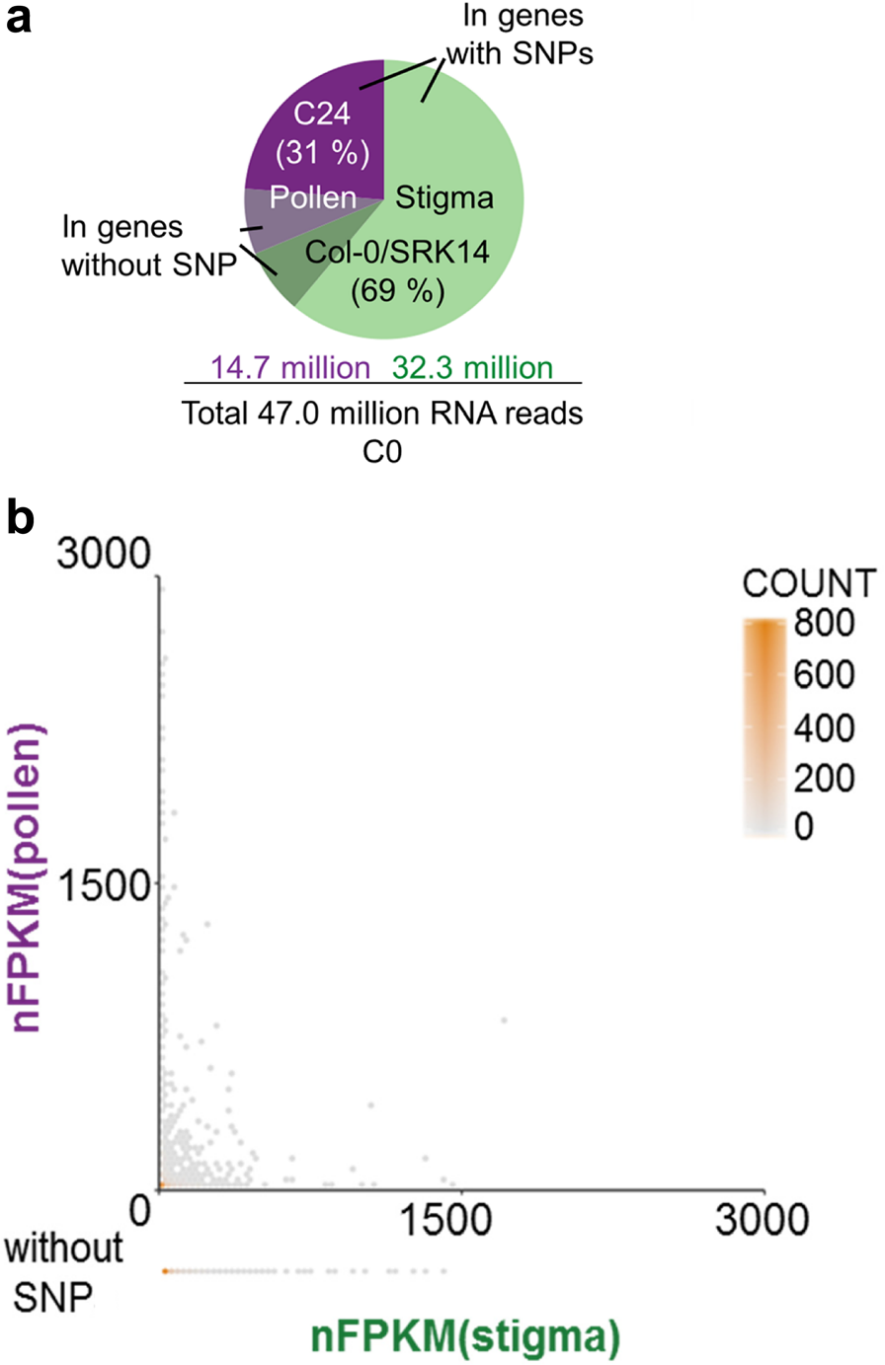
Quality check of SNP-based RNA-seq analysis. **a** Estimated proportions of reads allocated to each tissue (31% pollen, 69% stigma) including reads without SNP (15%) that were equally distributed between pollen and stigma (shaded green, 7.5% + shaded purple, 7.5%). **b** Hexbin plot to visualize distribution of gene expression in stigma (nFPKM stigma) and pollen (nFPKM pollen) at C0. Gene count per hexagon is represented using a color gradient from light grey to orange. Genes without SNP are plotted outside the graph based on their stigma expression (nFPKM stigma)

From this large dataset, we first determined the contribution of each tissue (stigma vs. pollen) in mixed samples harvested immediately after compatible pollination (C0). We found that among the 47.0 million RNA reads, ASE-TIGAR assigned only 15% reads to genes without SNPs. These reads were distributed equally between Col-0/SRK14 and C24 genomes using the Bayesian statistic. Thereby, 69% of the total RNA reads were estimated to derive from Col-0/*SRK14* (stigma), and 31% from C24 (pollen) (Fig. 2a). These proportions were stable over time (0, 10 min, 60 min) and independent of the pollination type (compatible, incompatible); this may reflect the relative abundance of stigmatic cells compared with pollen grains in our collected samples.

Then, we analyzed the relative abundance of each transcript between stigma and pollen. To do so, transcript abundance was computed by ASE-TIGAR and was expressed as Fragments Per Kilo base of exon per Million reads mapped (FPKM), which account for sequencing depth and gene length. We normalized FPKM (nFPKM) by dividing the FPKM of each transcript by the ratio of the transcript counts from stigma [nFPKM (stigma)] or pollen [nFPKM (pollen)] at C0 (Fig. 2a, Additional file 2: Table S3). Values of nFPKM were displayed on a hexbin [25] plot to visualize the distribution of gene expression levels. Genes without SNP did not show any particular pattern (Fig. 2b, bottom line). Gene expression levels were widely distributed, but almost all highly expressed genes were very specific to female or to male, suggesting the existence of distinct transcript signatures between stigma and pollen (Fig. 2b).

### Post-validation of the SNP-based analysis

**Fig. 3:**
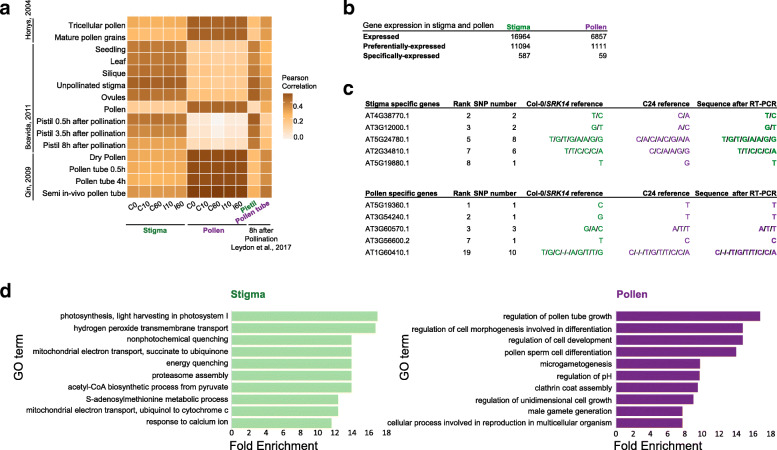
Validation of the SNP-based analysis. **a** Heat map of Pearson’s correlation coefficient between the stigma / pollen transcripts from the SNP-based analysis and transcript information from previously published data**. b** Number of stigma or pollen (preferentially / specifically) -expressed genes at C0 selected by nFPKM (Additional file 3: Table S4). **c** RT-PCR and sequence analysis of stigma or pollen specifically-expressed genes at C0. Genes are selected among the top 20 specifically-expressed genes; their rank according to their expression level are presented. RNAs were extracted from pollinated stigmas at C0 and we analyzed their SNP-information by RT-PCR and sequencing. SNP number corresponds to the number of SNPs present in the sequenced regions. **d** Top ten GO term enrichment categories (biological processes) of stigma or pollen preferentially-expressed genes at C0. Enrichment analysis was performed with the one thousand top expressed genes in stigma (left) or pollen (right), respectively. Selection criteria for genes analysed in **c** (sex-specific), and **d** (sex-preferential), are described in text

To assess the global consistency of our datasets with already published studies, we compared the results of our SNP-based expression analysis with tissue-specific transcripts reported from microarray experiments [26–28]. Stigma-associated transcripts from our analysis showed the highest correlation with transcriptome from unpollinated stigmas, and only a weak correlation with transcriptomes from mature pollen or growing pollen tube (Fig. 3a). Conversely, our pollen-associated transcriptome showed a very high correlation with male transcriptomes and almost no correlation with female transcriptomes or transcriptomes from vegetative tissues (Fig. 3a). Correlations between another SNP-based analysis, which identified pistil- and pollen tube-specific transcripts 8 h after pollination [19] showed similar trends to our stigma and pollen transcripts (Fig. 3a, two rightmost columns).

To have a global view of expressed genes in stigma and pollen at C0, we constructed three classes of genes from calculated nFPKM: expressed genes, sex-preferentially and sex-specifically expressed genes (see Methods for precise criteria of gene selection) (Additional file 3: Table S4). Briefly, we defined genes that showed nFPKM > 1 as expressed genes. A total of 21,335 genes were expressed in at least one condition; 16,964 and 6857 genes were expressed in stigma and pollen respectively (Fig. 3b). Genes that were expressed at least a ten-fold higher in stigma than in pollen were defined as stigma-preferentially expressed genes, and those that were at least one hundred-fold higher expressed in stigma than in pollen as stigma-specifically expressed genes (and vice versa for pollen preferentially and specifically expressed genes) (Fig. 3b, Additional file 3: Table S4). We first focused on sex-specifically expressed genes and analyzed the top 20 from stigma and pollen gene lists (Additional file 3: Table S4) using the ThaleMine database (https://apps.araport.org/thalemine/begin.do). Heatmaps of gene expression levels based on Cheng et al., 2017 [29] were generated (Additional file 1: Fig. S1). Stigma genes were clearly excluded from pollen even though many of them were also expressed in various tissues. By contrast, most pollen genes showed expression restricted to pollen and stage 12-inflorescences, which contain mature pollen. This result is consistent with the expression pattern that we observed on the hexbin plot (Fig. 2b). Moreover, within the top 20 stigma genes, we found seven of the 11 top genes ranked by expression levels in stigma RNA-Seq and microarray datasets [30], and as the top 1 pollen gene, we found *CPK34* (*AT5G19360*), the protein product of which is involved in pollen tube regulation [31]. Finally, to confirm the specificity of expression of these genes in stigma or pollen, we carried out RT-PCR and sequenced the cDNAs of SNP-containing regions using C0 sample as template. From the top 20 specifically expressed genes, we selected genes whose location of SNPs permitted designing of primers. As expected, based on the SNPs identified in their sequence, we found that mRNAs from stigma-specific genes came only from Col-0/*SRK14* (stigma), whereas mRNAs from the pollen-specific genes emanated only from C24 (pollen) (Fig. 3c).

To further characterize the pollen vs. stigma associated transcripts, we looked for Gene Ontology (GO) term enrichment at C0 within the list of sex-preferentially expressed genes (False Discovery Rate, FDR < 0.05) [32, 33]. Although GO terms may be somewhat subjective or not fully consolidated by functional tests, with the current annotation, we observed a clear difference between the two sets of transcripts (Fig. 3d). From GO term enrichment of the top 1000 preferentially expressed genes either in stigma or pollen (Additional file 3: Table S4, Additional file 4: Table S5), our analysis revealed high enrichment of several GO terms associated with metabolism in stigmas (such as “photosynthesis”, “mitochondrial-”, and “-metabolic process”) suggesting an active metabolic state of stigmatic cells (Fig. 3d left). Conversely, GO terms on the pollen side were specific to pollen functions such as “pollen tube growth”, “pollen sperm cell differentiation” and “cell tip growth” (Fig. 3d right).

The transcriptome of unpollinated stigmas was previously reported through laser microdissection of Arabidopsis stigmatic cells [32]. Among the top 100 expressed genes in this analysis, 44 were common with the top 100 expressed genes in stigma at C0, whereas no common genes were found with the top 100 expressed genes in pollen at C0.

Altogether, these results validate our SNP-based workflow, which allows identification of female- and male-derived transcripts from a combination of tissues following pollination.

### Gene expression dynamics triggered after pollination

**Fig. 4:**
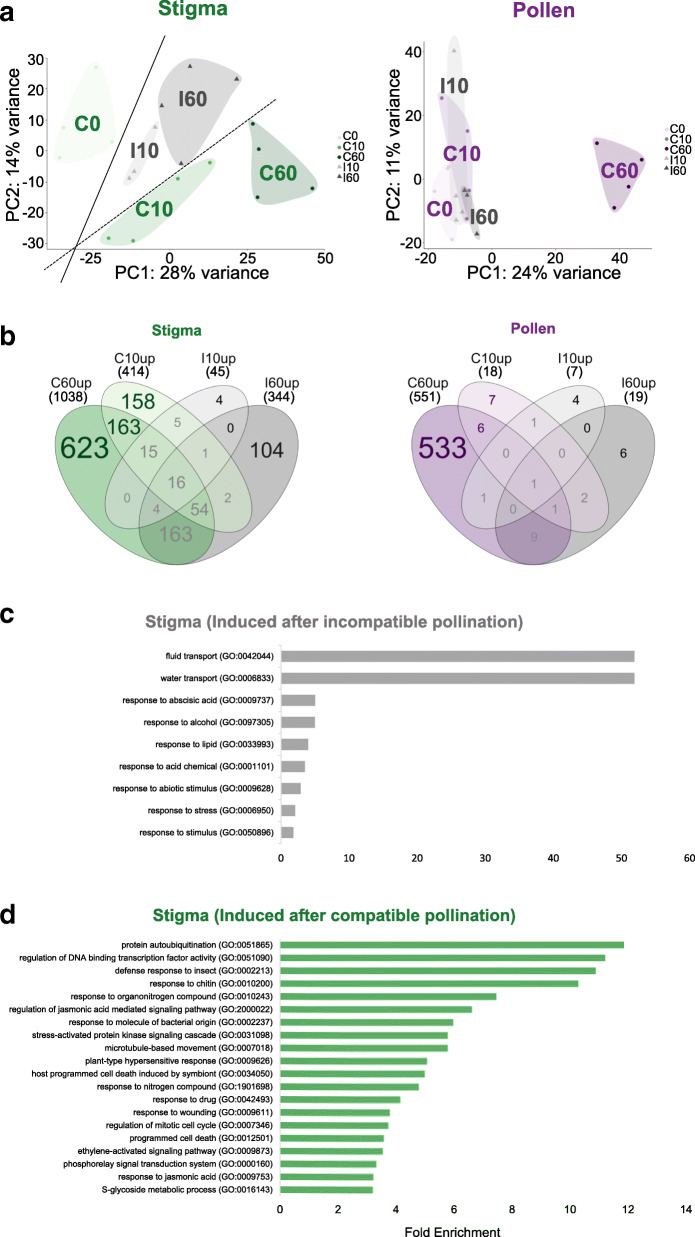
Gene expression dynamics after pollination. **a** PCA of stigma (left) and pollen (right) transcripts. Biological replicates (dots or triangles) in all pollination conditions were analyzed. **b** Venn diagrams showing the number of upregulated (FC > 2) genes in stigma (left) and pollen (right), after compatible (green and violet) or incompatible (grey) pollinations. **c** Gene enrichment categories of up-regulated genes after incompatible pollination in stigma according to GO terms of biological processes. Genes exclusively induced after incompatible reaction (b, left; 4 + 104) were analyzed. Only enrichment categories with significant FDR (< 0.05) are shown. d Gene enrichment categories of up-regulated genes after compatible pollination in stigma according to GO terms of biological processes. Genes exclusively induced after compatible reaction (b, left; 158 + 163 + 623) were analyzed. Only top 20 enrichment categories are shown

To examine the transcriptomic response of pollen and stigma following compatible or incompatible pollination, we first performed a principal component analysis (PCA) [34, 35] using the gene expression levels of each biological replicate in all conditions (Fig. 4a).

Expression levels in stigma and pollen were treated separately (Fig. 4a left and right, respectively). The total explained variance of the first two principal components (PC1 and PC2) is around 42% (28% + 14% respectively) for stigma and 35% (24% + 11% respectively) for pollen. The following two principal components (PC3 and PC4) are around 19% (10% + 9% respectively) for stigma and 15% (8% + 7% respectively) for pollen. Comparing the PCA of stigma and of pollen transcripts suggests different dynamics in each tissue. On the stigma side, the PCA shows that both compatible and incompatible pollinations triggered a transcriptional response, as along the PC1 axis, capturing almost 30% of the explained variance, samples are temporally sorted from 0 min to 60 min, thus suggesting that PC1 can be interpreted as a time axis. For the second axis, explaining 14% of the observed variability, compatible samples are located in the lower part whereas incompatible samples are located in the upper part, close to those of the starting point (C0). This seems to indicate that this axis is oriented by the compatible/incompatible effect. Meanwhile, on the pollen side, all the samples except C60 cluster together. Again, PC1 seems to capture the temporality of the compatible response, however we cannot conclude that PC2 is related to the compatible response effect since early compatible and incompatible clusters overlap on this axis. The clear response pattern of pollen in C60 samples is consistent with the massive changes displayed by compatible pollen after 1 h, which hydrated and germinated a pollen tube growing in the stigmatic tissue.

To get a quantitative view of the number of genes whose expression was modified during the course of pollination, we performed a differentially expressed gene (DEG) analysis (FC, Fold Change more than 2 between one condition and the zero-time point, padj < 0.1; Additional file 2: Table S3). We found more genes that were upregulated in at least one condition than downregulated (Additional file 1: Table S6). Then, we used a Venn diagram representation only with upregulated genes (FC > 2, padj < 0.1) to access the dynamics of gene expression upon pollination (Fig. 4b, Additional file 5: Table S7). The Venn diagram of upregulated genes in stigma (Fig. 4b left) showed an induction of gene expression in incompatible reactions, with 45 genes at I10 (corresponding to 0.2% (45/21335) of the total expressed genes) and 344 genes at I60 (1.6%). By comparison, a massive and progressive change of gene expression, following compatible pollination was detected, with 414 upregulated genes at C10 (2%) and 1038 (4.9%) at C60 (Fig. 4b left). On the pollen side (Fig. 4b right), very few genes showed altered expression, except in condition C60, where 551 genes were upregulated (2.6%). This correlates well with the dynamics of gene expression detected by PCA (Fig. 4a). Altogether, our data show a molecular reprogramming in stigma in response to both incompatible and compatible pollination, although more moderate in incompatible stigma. Meanwhile, compatible pollen grains reactivate their transcriptomic activity following germination and pollen tube growth in papilla cells, whereas the incompatible pollen remains almost quiescent.

### Pattern-triggered immunity (PTI) pathway induced after compatible pollination in stigma

To investigate the molecular events occurring during pollination, we applied a GO term enrichment analysis to genes that were exclusively upregulated in stigma after incompatible (104 + 4 = 108) and compatible (158 + 163 + 623 = 944) pollination (Fig. 4b left). In the case of the incompatible reaction, we found only 9 GO terms with high confidence (FDR < 0.05) (Additional file 6: Table S8, Fig. 4c). Nevertheless, it is worth noting that the two upmost enriched GO categories are related to water or fluid movements (water transport (GO:0006833), fluid transport (GO:0042044)). As Pollen hydration is highly controlled at the surface of Brassicaceae stigma [3], we may assume that these GO term categories reflect the regulation of water fluxes from the stigmatic cells towards the incompatible pollen.

At the opposite, after compatible pollination, there were 126 terms with high confidence (FDR < 0.05) among them, 85 had a very high confidence (FDR < 0.005) (Additional file 6: Table S8). These GO terms and enrichment were dramatically different from those preferentially expressed at C0 (Fig. 3d left, Fig. 4d). Among the top 20 upmost enriched categories, we found many GO terms related to defense response (defense response to insect (GO:0002213), response to chitin (GO:0010200), response to molecule of bacterial origin (GO:0002237), plant-type hypersensitive response (GO:0009626), host programmed cell death induced by symbiont (GO:0034050). Besides, we also found several GO categories related to wounding (regulation of jasmonic acid mediated signaling pathway (GO:2000022), response to wounding (GO:0009611), ethylene-activated signaling pathway (GO:0009873), response to jasmonic acid (GO:0009753)). The abundance of these GO term categories is likely to reflect the response of stigmatic cells to damages/stresses caused by the pollen tube while growing in the female tissues.

**Fig. 5:**
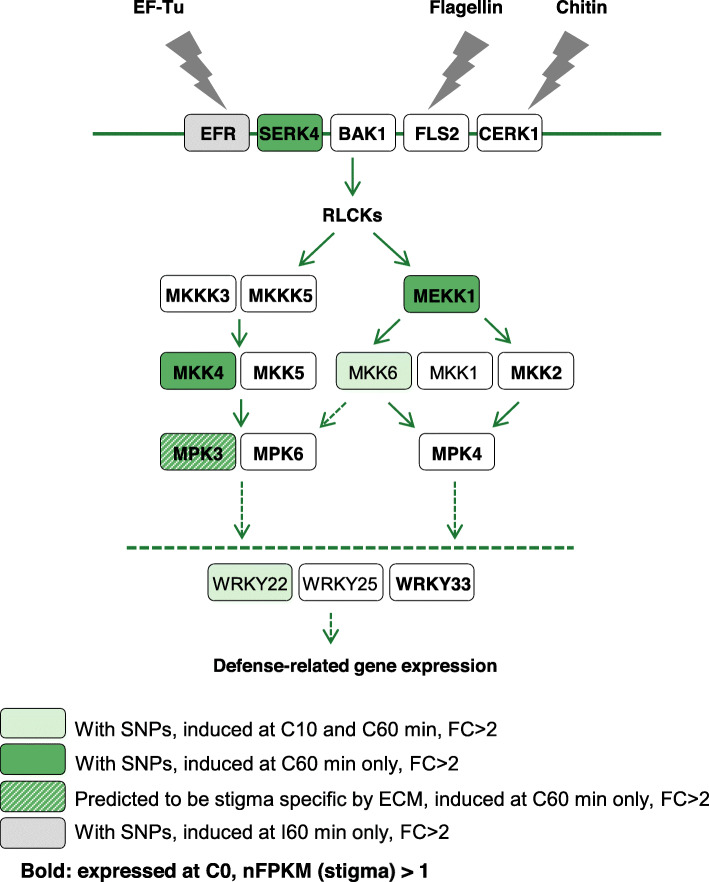
PTI pathway predicted to be induced in stigma after KEGG pathway mapping analysis. PTI pathway modified according to the information from Bigeard et al. 2015, Bi et al. 2018, and Lian et al. 2018. Genes in green boxes were upregulated (FC > 2) after compatible pollination, at 10 min and 60 min (light green), or only at 60 min (dark green: genes with SNPs, hatched dark green: genes without SNP but predicted stigma specific with ECM, see Supplementary file 8 Table S10). Gene in grey box was upregulated (FC > 2) 60 min after incompatible pollination. White boxes mean genes without SNPs (with no ECM prediction), not induced, or induced after both (compatible/incompatible) pollination (see Supplementary file 7 Table S9). Genes in bold were expressed at C0; nFPKM (stigma) > 1. RLCKs = Receptor-Like Cytoplasmic Kinases

The rapid and global transcriptomic changes in stigma after compatible pollination motivated us to search for molecular pathways activated. To predict such pathways, we mapped the 944 upregulated genes to KEGG pathways [35]. We found many metabolism-related pathways, signaling pathways such as hormone-signaling, plant-pathogen interaction and MAPK pathways. Interestingly, the plant-pathogen interaction pathway known as PTI induced by bacterial flg22 and EF-Tu [36] was predicted with 5 sequentially upregulated genes (SERK4, MEKK1, MKK4, MKK6, WRKY22, with FC between 2.2 and 2.9) in compatible situation but not in incompatible situation (Fig. 5, Additional file 7: Table S9), and among them, two genes (MKK6 and WRKY22) were rapidly induced from 10 min. One gene of this pathway, the receptor-like kinase EF-Tu Receptor (*EFR*) involved in bacterial pathogen associated molecular patterns (PAMP) was upregulated at I60 (FC = 2.2, padj = 1E-06). We cannot rule out that this might be biologically relevant in incompatible response. It is worth noting that *SERK4*, *BAK1*, *EFR*, *FLS2*, *CERK1*, all genes known to encode receptor-like kinases involved in plant-pathogen responses, were expressed preferentially in stigma at C0 (Additional file 3: Table S4), and among them, *SERK4* was upregulated at C60 (FC = 2.3) (Fig. 5, Additional file 7: Table S9).

To predict tissue specificity of genes without SNP, we developed a method based on gene expression correlation using the ATTED-II database (http://atted.jp/) [16, 17, 37] (see methods). Using this Expression Correlation Method (ECM), additional genes were predicted to be stigma specific (Additional file 8: Table S10). Interestingly, *MPK3* and *WRKY33* two hallmarks of the PTI pathway, were predicted to be stigma specific and were highly induced following compatible (FC = 6.3 and 10.0, respectively) compared with incompatible (FC = 1.5 and 2.8, respectively) reaction (Additional file 7: Table S9). Altogether, these analyses indicate that the PTI pathway is rapidly induced in stigma only after compatible pollination.

### Selectively upregulated genes after pollination in stigma and in pollen

**Fig. 6:**
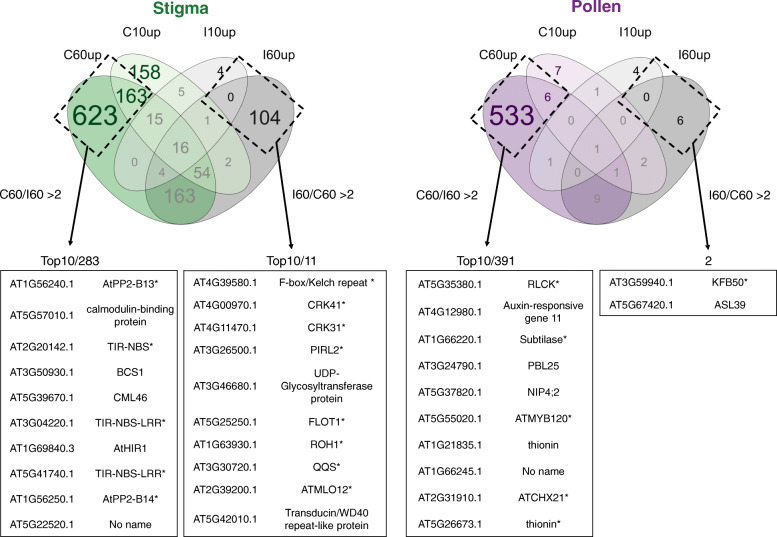
Selectively induced genes after pollination. From gene lists generated using a Venn diagram representation (Fig. 4b) we identified genes selectively induced 60 min after pollination in stigma (left) and in pollen (right) with criteria applied to the FC ratio between compatible and incompatible situations. The uppermost selectively induced genes sorted by FC ratio from the largest to the smallest (up to 10) are shown. * genes described in the discussion section

To identify new molecular players involved in pollen-stigma interaction, we strengthened our criteria to reduce potential candidates by retaining only genes exclusively upregulated after pollination (108 genes in incompatible situation, 944 genes in compatible situation; Venn diagram Fig. 4b, Additional file 5 Table S7) and sorted according to their fold change ratio between both pollination conditions (see methods for precise criteria). With these criteria, almost no gene were selectively induced 10 min after pollination and led us to focus on the 60-min pollination time (Fig. 6, Additional file 9: Table S11). In response to compatible pollen, we found five genes related to pathogen defense processes in the top 10 stigma genes (see discussion) which is in accordance with our KEGG pathway analysis. On the pollen side, several induced genes were already reported as pollen grain and/or pollen tube expressed genes (see discussion), underlining the reliability of our approach in discriminating pollen versus stigma transcripts. In the incompatible pollination, the uppermost upregulated genes in stigma and pollen were clearly distinct from those induced following pollen acceptance (see discussion), confirming the specific transcriptional changes linked to compatibility and incompatibility responses.

## Discussion

### Novel pipeline to analyse sex-specific transcriptome

To decipher the mechanisms that control reproduction in flowering plants, we extracted the global transcriptomic signatures associated with both pollen and stigmatic cells following their interaction during compatible or incompatible pollination. Although transcriptomes of pollinated stigmas have been recently published [16, 17], these analyses did not permit the distinction between pollen and stigma transcripts. In the present work, we provide a comprehensive transcriptomic dataset for stigma-pollen interactions using a novel SNP-based analysis. We took advantage of a recently developed statistical tool called ASE-TIGAR, which is based on a Bayesian approach to estimate allele-specific expression in diploid cells [20]. Nariai et al. showed an accurate estimation of gene expression from RNA-seq with ASE-TIGAR and identified some autosomal genes as allele-specific genes in a human reference lymphoblastoid cell line. We applied this new tool to discriminate transcripts from female-male mixed tissues during the first steps of the fertilization process. Our experimental design and bioinformatic pipeline allowed us to unveil the transcriptomic response of the pollen/pollen tube and that of the stigma following compatible or incompatible pollination at two early (10 min and 60 min) time points of the male-female interaction (Fig. 1). It is worth noting that another SNP-based pipeline has recently been published [19], identifying transcripts derived from the pistil and pollen tubes collected 8 h after compatible pollination. Although we cannot compare the two analyses directly, as pollination time points were different and only compatibility was studied by Leydon et al., both analyses succeeded in distinguishing male and female transcripts as presented in Fig. 3. However, while Leydon et al. used 12% of reads that had SNPs among the total sequenced reads, in our analysis we took all the RNA-seq reads into account, including those without SNP [19, 20]. This difference in the approaches has likely contributed to making our analysis more exhaustive, as exemplified by the remarkable segregation we found between male transcripts and those derived from female and vegetative tissues (Fig. 3a). In addition, the new expression correlation method (ECM) we developed here, allowed us to predict sex-specificity for some genes without SNP. The sex preferentially- or specific-expressed gene lists we drew up contain many of the already identified female or male specific genes and reported GO term enrichment [8, 11, 13, 30, 32] (Fig. 3d). Together with the correlation analysis (Fig. 3a) and experimental evaluations (Fig. 3c), we can conclude that our female- / male-transcripts globally represent stigma−/pollen-transcripts.

### Global molecular dynamics after compatible and incompatible pollination

While the Venn diagram (Fig. 4b) shown that transcriptional activity occurs after pollination, the percentage of upregulated genes remains however low, varying from 0.2 to 4.9%. This is in accordance with another transcriptomic analysis in Arabidopsis [16], which reported only a few percent of difference between transcripts before and 60 min after pollination for both compatible and incompatible interactions.

Even though our data are consistent with other transcriptomic analyses of pollinated stigmas [8, 16, 32], the clear distinction we made between male and female transcripts also reveals unanticipated characteristics (Figs. 3 and 4). The Venn diagram suggested a rapid transcriptomic response in the stigma for compatible (with 231 genes exclusively induced at C10) but not for incompatible pollination (with only 4 exclusively upregulated genes at I10) (Fig. 4b left). This suggests that key molecules required for the incompatibility reaction are likely to be already present in the stigma. This may be consistent with the observation that the pollen rejection response in *A. thaliana* lines exhibiting a restored incompatibility system is extremely rapid and occurs within minutes [3, 12].

While no cellular changes were detectable by SEM on stigmas 1 hour after incompatible pollination (Fig. 1a), we identified more than one hundred genes exclusively upregulated in stigmas at I60 (104 genes, Fig. 4b left). This demonstrates that the stigma undergoes molecular changes following incompatible pollination. As the incompatibility reaction is maintained for several days, at least as long as SRK is properly expressed in the stigma, we may propose that among the stigma upregulated genes there are key factors required for proper development of the SI response and its maintenance over time.

### Plant responses to pollen and pathogens share conserved molecular mechanisms

The similarity between the stigma-pollen interaction and plant-pathogen interaction has been discussed and has drawn researchers’ attention for a long time [10, 38–40]. Several transcriptomic analyses of pollinated stigmas/pistils reported enrichment in transcripts linked to stress or defense response in female tissues [19, 27, 40–42] and it was suggested that the self-incompatible response shared common mechanism with plant-pathogen interaction as it is a response to block the invader. Based on a time course transcriptome analysis of compatible and incompatible reactions in *Brassica napus*, Zhang and collaborators speculated that both compatible and incompatible reactions had close parallels with plant-pathogen interactions. Although we found some upregulated defense related genes in the incompatible reaction (see below), KEGG analysis indicates that components of PTI pathway were induced only during compatible response. (Fig. 5, Additional file 7: Table S9). This result was not completely unexpected since pollen tube growth resembles pathogen attack from several angles, for example invasive growth of an external organism, cell wall digestion, and calcium burst [8, 43, 44]. However, it is unclear why PTI pathway, whose function is to block the pathogen development, is induced in the stigma while pollen tube growth is promoted. We may suppose that components of this PTI pathway participate in the acceptance of pollen grains. Another alternative would be that penetration of the pollen tube in the papilla cell wall provide a route of entry for bacterial and fungal pathogens and hence, the PTI response would prevent infection by pathogens during compatible pollination.

### Upregulated genes induced after SC response in pollen and stigma

Among the top 10 genes selectively upregulated 1 hour after compatible pollination in stigma (Fig. 6, Additional file 9: Table S11), we found the two closely related genes, *AT1G56240* and *AT1G56250* encode proteins containing a lectin domain, known to bind specific carbohydrate structures [45] and a F-box domain. F-box proteins have been reported to be components of the SCF (SKP1-CUL1-F-box protein) ubiquitin ligase complex, where they mediate polyubiquitination of target proteins for their subsequent proteasome-dependent degradation [46]. The three others upregulated genes related to plant defense, encode a product with a Toll/interleukin-1 receptor (TIR) domain (*AT2G20142, AT3G04220, AT5G41740*, Fig. 6, Additional file 9: Table S11). The Arabidopsis genome contains 135 genes encoding proteins with a TIR domain that could be associated with others motifs, including nucleotide-binding site (NBS) and leucine-rich repeat (LRR), [47]. Association of TIR-NBS-LRR domains is found in several disease resistance (R) protein involved in pathogen recognition [48] and, one R-genes is upregulated in response to pollen recognition (*AT5G41740)*.

The highest upregulated gene in pollen, selectively induced 60 min after compatible pollination (Fig. 6, Additional file 9: Table S11), with a very high fold change induction compared with incompatible pollination (> 100 fold) is *AT5G35380*. The encoded protein belongs to the Receptor-Like Cytoplasmic protein Kinase family (RLCK), whose members contain a domain with predicted kinase activity but lack an extracellular domain. Several RLCKs have been described as required for successful sexual reproduction and function downstream of kinase receptors [49]. Upstream of the kinase domain, the RLCK *AT5G35380* contains a domain with similarity to the bacterial Universal Stress Protein A (UPSA). In bacteria, UPSA is implicated in coordinating the metabolic transition observed when bacterial cells are growing on minimal medium with glucose as the sole carbon source [50–52]. UPSA domains have been found in 44 *A. thaliana* proteins but their functions in plant cells remain enigmatic [53]. We may postulate that increased levels of *AT5G35380* during pollen germination and early pollen tube growth could be involved in the metabolic transition from a quiescent desiccated pollen grain towards an active germinating grain and growing pollen tube in coordination with an as-yet uncharacterized kinase receptor.

Among the others pollen genes, we found the transcription factor AtMYB120 (*AT5G55020*) that, together with two closely related MYBs (AtMYB97 and AtMYB101), is required for pollen tube growth arrest and sperm cell release [54]. These three MYB factors regulate the expression of a set of pollen tube genes [19, 54] among them, one plant-specific antimicrobial peptides from the thionin family (*AT5G26673*) and one Subtilisin-like protease (*AT1G66220*), which are both present in our top 10 list. We also found increased expression of the K^+^-transporter /proton exchanger gene *CHX21* (*AT2G31910*), the corresponding protein localizes to the endoplasmic reticulum of pollen tubes and is involved in the reorientation of pollen tube growth towards the ovules (Fig. 6, Additional file 9: Table S11) [55]. Altogether, our data suggest that genes described as involved in late stage of pollen tube guidance within the pistil tissues, may also have roles in earlier stages of male-female interaction and could function in guiding the pollen tube throughout its journey in the stigma/style.

### Upregulated genes in pollen and stigma following SI response

Astonishingly, the two highest upregulated genes in the pollen and stigma after 1 hour of incompatible pollination belong to the same family of genes and encode F-box/Kelch repeat proteins (Fig. 6, Additional file 9: Table S11). F-box/Kelch repeat proteins are involved in various processes such as response to biotic [56–58] and abiotic stress [59, 60] hormone signaling [61], metabolic pathways [62–65], cell expansion [66], and circadian clock regulation [67–69]. The F-box/Kelch repeat protein gene *KFB50/KMD4* (*AT3G59940*), has three homologs designated *KMD1* (*AT1G80440*), *KMD2* (*AT1G15670*) and *KMD3* (*AT2G44130*) [61]. All four encoded proteins have redundant functions in negatively regulating cytokinin response by degrading type-B Arabidopsis response regulators (ARRs) [61]. Interestingly, analysis of triple mutants for the three cytokinin receptors (CRE1, AHK2 and AHK3) revealed that cytokinin perception is essential for pollen function as *cre1 ahk2 ahk3* pollen, while capable of germinating in vitro, cannot germinate on wild-type stigmas [70]. Based on these data, we may suggest that the increased expression of *KFB50/KMD4* in incompatible pollen grains impairs cytokinin response and hence inhibits germination. How the signaling cascade initiated in the papilla cell following SCR-SRK interaction may induce changes in *KFB50/KMD4* expression in the pollen remains yet elusive. In the stigma, the F-box/Kelch repeat protein gene *AT4G39580* is strongly induced (FC ~ 7) following SI reaction (Additional file 9: Table S11). In Brassica sp. and *A. lyrata*, SI response in the stigma has been associated with the activation of a U-box/ARM-repeat E3 ligase (ARC1) that is believed to degrade compatibility factors required for pollen germination and tube growth [71]. Although *A. thaliana* does not have a functional *ARC1* gene [72], co-expression of *B. napus ARC1* or *A. lyrata ARC1* along with *A. lyrata SCR-SRK* gene pair confers a strong SI response in transgenic *A. thaliana* [13]. Our work shows that SCR14-SRK14 interaction can induce a strong SI response in *A. thaliana* without the need of co-expressing *ARC1* [3]. We might suggest that the F-box/Kelch repeat protein AT4G39580 could substitute for ARC1 in the proteasome degradation pathway leading to self-pollen rejection.

The two other uppermost upregulated genes in stigmas are the cysteine-rich receptor-like protein kinase genes *CRK41* (*AT4G00970*) and *CRK31* (*AT4G11470*) (Fig. 6, Additional file 9: Table S11). In Arabidopsis, *CRKs* form a large family of 44 members that are involved in a broad variety of biological processes including plant growth and development as well as plant response to biotic and abiotic stresses [73]. Following activation of the receptor-like protein kinase FLAGELLIN-SENSING2 (FLS2) by its ligand flagellin (flg22), CRK28 was recently shown to physically interact with another receptor-like kinase (RLK), the BRASSINOSTEROID INSENSITIVE 1-associated receptor kinase1 (BAK1) and to associate with the FLS2-BAK1 immune complex [74]. In addition, CRK28 was also found to bind to the closely related CRK29. As abundance of these two CRKs increased after flg22 perception and that CRK28 overexpression enhanced disease resistance to *Pseudomonas syringae*, it is tempting to speculate that CRK41 and CRK31 might similarly interact with the activated SRK following SCR binding to regulate the SI signaling cascade. Among the other upregulated genes in the stigma, PIRL2 (*AT3G26500*) protein is involved in gametophyte development [75], FLOT1 (*AT5G25250*) in a clathrin-independent endocytosis pathway [76], the degradation of which is enhanced following flg22 elicitation [77], ROH1 (*AT1G63930*) in secretion of seed coat pectin and exocyst function [78], QQS (*AT3G30720*) in plant defense [79], and MLO12 (*AT2G39200*) in defense response to fungi as a susceptibility factor to powdery mildew pathogens [80] (Fig. 6, Additional file 9: Table S11). The functions of these SI upregulated genes are mostly related to cell signaling pathways and plant response to stress, and for some of them to cellular mechanisms that have already been described to operate in SI such as RLK signaling, proteasome-dependent degradation of proteins, exocyst-dependent secretion and endocytosis [81]. Internalization of SRK was reported in *Brassica oleracea* following self-pollination and this was associated with SRK degradation, suggesting that initiation of the signaling cascade occurs at the plasma membrane and that endocytosis of the activated receptor is involved in the downregulation of SI pathway [82]. Recently, clathrin-mediated endocytosis was shown to be unnecessary for SRK signaling [83]. Linked with this latter work, it is of particular interest to note an increased level of FLOT1 expression in our transcriptomic analysis. Indeed, FLOT1 protein is required for Sterol-Dependent Endocytosis (SDE) of membrane-associated proteins, which is another mode of endocytosis that works independently of clathrin [84]; besides, FLOT1 is degraded following internalization [77]. All together, we might hypothesize that endocytosis of the activated SCR-SRK complex would depend on SDE, and that neo-synthesis of FLOT1 would be necessary to fine-tune the SI response.

## Conclusion

During reproduction, the mixture of parental transcripts makes challenging the characterization of male- and female-specific patterns of gene expression. Our work, based on SNP-based RNA-seq analysis coupled with the statistical methodology ASE-TIGAR, allows us to distinguish the parental origin of 80% of transcripts and provides an accurate view of dynamic transcriptional changes occurring both in pollen and stigma following compatible and incompatible pollinations. We report hundreds of genes whose expression is upregulated during the compatibility response. Remarkably, KEGG prediction identifies components of PTI pathways that are activated in the stigma upon compatible pollination. We speculate that this defense response may contribute to the signaling cascade leading to pollen acceptance or, alternatively, may protect the pistil, which is invaded by hundreds of pollen tubes, from concomitant pathogen attacks. We also report the upregulation of a hundred genes in the stigma following self-pollen rejection, that are likely to participate in the regulation or maintenance of SI over time. These genes may have retained their functionality in *A. thaliana*, which is in agreement with the estimation of recent transition to selfing in this species [85, 86]. Overall, our work provides substantial resources and innovative tools to identify novel molecular players in the fertilization process.

## Methods

### Plant material and growth condition

*Arabidopsis thaliana* C24 (N22680) and Col-0 (N22681) seeds were obtained from NASC [87]. We generated transgenic plants like previously reported [3]. *AlSRK14* construct was introduced in Col-0 (Col-0/*SRK14*). *AlSCR14* construct was introduced in C24 (C24/*SCR14*). All plants were grown in growth chambers under long-day cycles (16 h light/8 h dark at 21 °C/19 °C). Transgenic seeds are available upon request.

### Environmental scanning electron microscopy (SEM)

Flowers from stages 12 were emasculated and pollinated on plants with mature pollen. One hour after pollination, pistils were cut in the middle of the ovary, deposited on a SEM platform and observed under Hirox SEM SH-3000 at − 20 °C, with an accelerating voltage of 10 kV. Images were processed with ImageJ software 1.53b version.

### Genomic DNA and mRNA preparation

For genomic DNA extraction, about 2 mL volume young inflorescences of Col-0/SRK14 and C24 were harvested, respectively. After grinding the material in liquid nitrogen, DNA was extracted as described previously [88]. For RNA extraction, late stage 12 [23] Col-0/SRK14 flowers were emasculated and 16-20 h after emasculation stigmas were pollinated with compatible (C24) or incompatible (C24/*SCR14*) pollen. 0, 10 or 60 min after compatible pollination (C0, C10 and C60) or incompatible pollination (I10, I60), 50 stigmas were harvested manually using fine tweezers, then frozen in liquid nitrogen and stored at − 80 °C until further processing. After grinding the material in liquid nitrogen, RNA was extracted and purified by using Arcturus® PicoPure® RNA Isolation Kit (Applied Biosystems / Thermo Fisher Scientific) following the manufacturer instruction except that we added a DNAse treatment (Qiagen, catalog#79254). Five biological replicates of RNA at each point were prepared, then four replicates were selected for sequencing based on their quality and quantity.

### Whole genome sequencing and variant calling

Library preparation and whole genome sequencing of Col-0/SRK14 and C24 were performed by HELIXIO (Clermont-Ferrand, France; http://www.helixio.com/) with TruSeq® DNA PCR-free Library Preparation kit (Illumina) and NextSeq500 platform (Illumina) applying paired-end sequencing (2 × 150 bp) (Additional file 1: Table S1). We then proceeded to the analysis of the sequencing data using GATK3.5 after quality check using FastQC (http://www.bioinformatics.babraham.ac.uk/projects/fastqc), trimming and pairing of the resulting reads using custom Perl scripts. We aligned the reads to the Col-0 public genome (TAIR10 public genome refers to the TAIR9 genome sequence and the TAIR10 annotation for Arabidopsis Col-0, which are both available from http://www.arabidopsis.org/. For simplicity, we refer to them as simply TAIR10 because no genome sequence changes were made between the TAIR9 and TAIR10 annotation releases) with BWA [89] applied GATK [90] base quality score recalibration, indel realignment, duplicate removal, and performed SNPs and indels discovery across all samples according to GATK Best Practices recommendations [91, 92]. After checking the depth of coverage of two samples by using GATK Depth Of Coverage (Additional file 1: Fig. S2), we performed filtering applying depth 3 for Col-0/SRK14 and depth 6 for C24 and homozygous for both, then obtained the complete set of variants for each genome as VCF files.

### Production of new reference genomes

Col-0/SRK14 and C24 genome sequences were derived from TAIR10 genome, by introducing the called variants (only SNPs and not indels) in the sequence, by using GATK Fasta Alternate Reference Maker. Gene annotations from TAIR10 genome were transferred onto the two new genome sequences using RATT [93] in “Strain” mode (a gene is annotated if it exhibits at least 95% similarity with the gene model) and seqret from EMBOSS suite [94]. To characterize polymorphism (only SNPs) between obtained Col-0/SRK14 and C24 genome sequences, inside and outside predicted genes, we aligned chromosome sequences using LAST (v. 938) [95], after training LAST on chromosomes 1.

### RNA sequencing and expression analysis

Library preparation and RNA sequencing were performed by HELIXIO with TruSeq® Standard mRNA sample Preparation kit (Illumina) and NextSeq500 platform (Illumina) applying paired-end sequencing (2 × 75 bp). We then proceeded to the analysis of the sequencing data using ASE-TIGAR after quality check by FastQC, trimming and pairing of the resulting reads using custom Perl scripts. First, we derived mRNA reference sequences from new reference genomes and merged them in one FASTA file, then mapped the clean reads on the mRNA reference with Bowtie2 [96]. We then run ASE-TIGAR with SAM files produced by mapping and the mRNA reference (http://nagasakilab.csml.org/ase-tigar/) [20].

Raw sequencing data is available on NCBI database under the SRA accession number SRP154565 (https://www.ncbi.nlm.nih.gov/sra/SRP154565).

### Differential gene expression analysis and calculation of nFPKM

Differential expression analysis on the whole transcriptome was performed using DESeq2 [24]. After performing principal component analysis (PCA) for all biological replicates at each condition, we removed one replicate at I10 from female samples and one at I60 from male samples, as they were isolated from the other replicates. All the analyses, including the presented PCA, were performed with the sample set after the removal.

Normalized FPKM (nFPKM) was calculated by dividing stigma- or pollen-FPKM by female or male transcript proportion at each condition (C0, C10, C60, I10, I60).

### Comparison with published datasets

To check the consistency of our RNA-seq data with previously published microarray data [26–28], for each pair of condition, we computed a Pearson correlation coefficient (r) over all genes with SNPs from our variant analysis, using mean of estimated read counts from three or four biological replicates for RNA-seq and mean absolute intensity for multiple microarray replicates. We also computed Pearson correlation coefficients (r) between previously published RNA-seq data [19] and the microarray data sets. Then, the heat map was drawn using an original R-script (R version 3.4.2) and libraries Hmisc, reshape2 and ggplot2.

### Criteria to select expressed genes, sex–preferentially or specifically expressed genes

Among genes with SNPs between Col-0 and C24/*SRK14*, we defined expressed genes, sex-preferentially or sex-specifically expressed genes at C0 (Fig. 3b, Additional file 3: Table S4), with criteria applied to nFPKM. We defined stigma-expressed genes as [nFPKM (stigma) > 1] and stigma-preferentially expressed genes as [nFPKM (stigma) > 1 ∩ nFPKM (stigma) ≥ 10 × nFPKM (pollen)]. Stigma-specifically expressed genes were selected among stigma-preferentially expressed genes applying more stringent criteria. Stigma-specifically expressed genes are defined as [stigma preferentially expressed genes ∩ nFPKM (pollen) ≤ 1 ∩ nFPKM (stigma) > 100 ∩ nFPKM (stigma) > 100 x nFPKM (pollen)], then sorted by nFPKM (stigma) from the largest to the smallest. Vice-versa for pollen (preferentially / specifically) expressed genes. The same criteria were applied for other pollination time-points (Additional file 8: Table S10).

### GO term and enrichment analysis and pathway analysis

GO and gene enrichment analyses were performed using Gene Ontology Consortium website (http://www.geneontology.org/) and PANTHER 13.1 software [97, 98]. We used “GO biological process complete” for the classification. GO terms with FDR < 0.05 were considered as significantly enriched.

Pathway analysis was performed using and KEGG PATHWAY Database using KEGG Mapper software (http://www.kegg.jp/kegg/tool/map_pathway2.html) [35].

### RT-PCR and sequencing

For experimental evaluation of the SNP-based analysis with reverse transcription polymerase chain reaction (RT-PCR) and sequencing, we selected genes among the top-20 specifically expressed genes that have SNPs at suitable positions allowing discrimination of parental origin (stigma vs. pollen) (Fig. 3c). RNA at C0 was purified as described above. cDNA was generated using SuperScript® VILO™ cDNA Synthesis Kit (Invitrogen). Primers were designed by Primer3web (http://bioinfo.ut.ee/primer3/) [99] at the flanking region of SNP including sites. PCR was performed with GoTaq (Promega). PCR products were purified using PCR clean-up Gel extraction kit (Macherey-Nagel) and then sequenced.

### Criteria to select induced genes in stigma and pollen after compatible/incompatible responses, based on FC

Among genes with SNPs between Col-0 and C24/*SRK14*, we selected genes which are induced after compatible and incompatible pollination in stigma or in pollen respectively with criteria applied to FC. We selected induced genes 10 min after compatible reaction in stigma as [FC_Col-0/SRK14_C10/C0 > 2] with padj < 0.1. We proceeded in the same way for 60 min time-point, for incompatible situation and for pollen (Additional file 5: Table S7).

Genes selectively upregulated in stigma after compatible pollination were selected among those exclusively upregulated after compatible pollination (Venn diagram Fig. 4b), applying criteria on FC Col-0/SRK14_C60/I60 > 2, with padj < 0.1, then sorted by FC_Col-0/SRK14_C60/I60 from the largest to the smallest. Vice-versa for incompatible situation and for pollen (Additional file 9: Table S11).

### Prediction of sex-specificity for genes without SNP between Col-0/*SRK14* and C24

To infer sex-specificity of genes without SNP between Col-0/*SRK14* and C24 but with high expression levels in our RNA-seq experiment, we used the database ATTED-II (http://atted.jp/) [37] to select genes with correlated expression patterns. Only genes with nFPKM (stigma+pollen) ≥ 350 were considered for specificity prediction, as we showed that most of these genes were sex-specific (Fig. 2b). Based on the genes having SNPs between the two accessions, this subset contains 86.3% sex-preferentially- or sex-specifically-expressed genes at C0. For each target gene, we retrieved its ATTED-II record from either the Ath-m v7.1 dataset (based on 16,033 microarray hybridizations, [37]) or, if not available, the Ath-r dataset v3.0 (based on 2120 RNA-seq experiments). Based on the mutual rank, we kept the 50 most highly correlated genes having polymorphism between Col-0/*SRK14* and C24, and we looked at their sex-specificity in our dataset. Using the 505 target genes with SNPs, we determined simple ad-hoc criteria to predict sex-specificity: target genes with expression pattern correlated with more than 35 stigma-specific genes were considered stigma-specific; target genes correlated with more than 25 pollen-specific genes were considered pollen-specific. With these thresholds, the specificity prediction was correct for 96.7% of stigma-specific genes and 97.8% of pollen-specific genes; 93 genes out of 505 remained without prediction and the prediction was wrong in only 12 cases (Additional file 8: Table S10). We applied the same criteria to the 161 target genes with no SNPs and nFPKM (stigma+pollen) ≥ 350, and we were able to predict stigma-specificity for 129 of them and pollen specificity for 28 genes (Additional file 8 Table S10). We named this method “Expression Correlation Method (ECM)”.

## Supplementary Information

**Additional file 1: Figure S1.** Known gene expression information for sex-specifically expressed genes. Top 20 sex-specifically expressed genes were analysed with ThaleMine database (https://apps.araport.org/thalemine/begin.do). Heat maps of RNA-seq based gene expression levels (Cheng et al., 2016) for each list were derived (stigma: upper, pollen: lower). * genes analyzed by RT-PCR and sequencing in Fig. 3c. **Figure S2.** Depth coverage of Col-0/*SRK14* and C24 reference genomes after variants calling. Distributions of depth coverage before filtering were obtained by using GATK Depth Of Coverage. After checking depth coverage, Col-0/*SRK14* variants were filtered at depth 3 and C24 variants were filtered at depth 6. **Table S1.** Read length and depth of whole genome sequencing. Col-0/SRK14 and C24 genomes were sequenced with NextSeq500 platform (Illumina) applying paired-end sequencing (2×150 bp). Sequence quality was checked by FastQC (http://www.bioinformatics.babraham.ac.uk/projects/fastqc). Read parts with quality score less than 26 were trimmed and reads shorter than 50bp were discarded. F = forward sequence, R = reverse sequence. **Table S2.** Length of sequenced RNA reads. Four RNA replicates selected from five samples at each pollination condition were sequenced with NextSeq500 platform (Illumina) applying paired-end sequencing (2×75 bp). Sequence quality was checked by FastQC (http://www.bioinformatics.babraham.ac.uk/projects/fastqc). Read parts with quality score less than 26 were trimmed and reads shorter than 40bp were discarded. F=forward sequence, R = reverse sequence. **Table S6.** Number of up- and down-regulated genes. Genes up-regulated (FC > 2.0, padj < 0.1) and down-regulated (FC < − 2.0, padj < 0.1) were selected.

**Additional file 2: Table S3.** Relative abundance of each transcript between stigma and pollen

**Additional file 3: Table S4.** Expressed genes, sex-preferentially and sex-specifically expressed genes.

**Additional file 4: Table S5.** GO term enrichment of the top 1000 preferentially expressed genes in stigma or in pollen

**Additional file 5: Table S7.** Genes analyzed with a Venn diagram representation

**Additional file 6: Table S8.** GO term enrichment of genes induced after pollination in stigma

**Additional file 7: Table S9.** Genes in the PTI pathway induced in stigma after compatible pollination.

**Additional file 8: Table S10.** Prediction of sex-specificity by ECM

**Additional file 9: Table S11.** Selectively induced genes after compatible/incompatible responses in stigma or pollen.

## Data Availability

The datasets used and/or analyzed during the current study are available from the corresponding authors on reasonable request.

## Abbreviations

SI: Self-incompatibility
S-locus: Self-incompatibility-locus
SCR: S-locus Cysteine Rich protein
SP11: S-locus protein 11
SRK: S-locus Receptor Kinase
ATPase: Adenosine triphosphatase
EXO70A1: Exocyst complex component 70 A1
RNA: Ribonucleic acid
DNA: Deoxyribonucleic acid
SNP: Single nucleotide polymorphism
ASE-TIGAR: Allele-specific expression-transcript isoform abundance estimation with gapped alignment of RNA-seq data
PTI: Pattern-triggered immunity
Indels: Small insertions and deletions
TAIR: The Arabidopsis information resource
UTR: Untranslated region
CDS: Coding sequences
DESeq: Differential expression analysis for sequence count data
FPKM: Fragments per kilo base of transcript per million reads mapped
nFPKM: Normalized FPKM
Hexbin: Hexagonal binning
CPK34: Calcium-dependent protein kinase 34
RT-PCR: Reverse transcriptase-polymerase chain reaction
FDR: False discovery rate
GO: Gene ontology
PCA: Principal component analysis
PC: Principal components
DEG: Differentially expressed gene analysis
FC: Fold change
Padj: Adjusted *p*-value
SERK: Somatic embryogenesis receptor kinase
MEKK: Mitogen-activated protein kinase kinase kinase
MKK: Mitogen-activated protein kinase kinase
WRKY: WRKYGQK containing transcription factor
RLK: Receptor-like kinase
EF-Tu: Elongation factor thermo unstable
*EFR*: EF-Tu receptor
PAMP: Bacterial pathogen associated molecular patterns
BAK1: Brassinosteroid insensitive 1-associated receptor kinase 1
Flg-22: Flagellin
FLS2: Flagellin-sensing 2
CERK1: Chitin elicitor receptor kinase 1
ATTED-II database: *Arabidopsis thaliana* trans-factor and cis-element prediction database
ECM: Expression correlation method
*MPK*: Mitogen-activated protein kinase
KEGG: Kyoto encyclopedia of genes and genomes
SEM: Scanning electron microscopy
SCF: SKP1-CUL1-F-box protein
TIR: Toll/interleukin-1 receptor
NBS: Nucleotide-binding site
LRR: Leucine-rich repeat
RLCK: Receptor-like cytoplasmic protein kinase
UPSA: Universal stress protein A
MYB: Myeloblastosis
*CHX21*: Cation/H+ exchanger 21
*KFB*: Kelch repeat F-box
KMD: Kiss me deadly
ARR: Type-B Arabidopsis response regulators
CRE1: Cytokinin receptors 1
AHK: Histidine kinase homologs
ARC1: Armadillo repeat containing 1
CRK: Cysteine-rich receptor-like protein kinase
PIRL2: Plant intracellular ras-group-related LRR 2
FLOT1: Flotillin-like protein 1
ROH1: Czech roh meaning corner 1
QQS: Qua-quine starch
MLO: Barley mildew resistance locus o
SDE: Sterol-dependent endocytosis
NASC: Nottingham Arabidopsis stock centre
GATK: Genome analysis toolkit
FastQC: Fast quality control
BWA: Burrows–Wheeler-alignmer
VCF: Variant call format
MCBI: National center for biotechnology information
RATT: Rapid annotation transfer tool
EMBOSS: European molecular biology open software suite
LAST: Genome-scale sequence comparison
SAM: Sequence alignment map
