# Supplementary material for: The molecular signatures of compatible and incompatible pollination in Arabidopsis
Source: BMC Genomics. 2021 Apr 14;22:268. doi: 10.1186/s12864-021-07503-7 (PMC8048354; doi:10.1186/s12864-021-07503-7)
Supplement: Supplementary file 1 — Additional file 1: Figure S1. Known gene expression information for sex-specifically expressed genes. Top 20 sex-specifically expressed genes were analysed with ThaleMine database (https://apps.araport.org/thalemine/begin.do). Heat maps of RNA-seq based gene expression levels (Cheng et al., 2016) for each list were derived (stigma: upper, pollen: lower). * genes analyzed by RT-PCR and sequencing in Fig. 3c. Figure S2. Depth coverage of Col-0/SRK14 and C24 reference genomes after variants calling. Distributions of depth coverage before filtering were obtained by using GATK Depth Of Coverage. After checking depth coverage, Col-0/SRK14 variants were filtered at depth 3 and C24 variants were filtered at depth 6. Table S1. Read length and depth of whole genome sequencing. Col-0/SRK14 and C24 genomes were sequenced with NextSeq500 platform (Illumina) applying paired-end sequencing (2×150 bp). Sequence quality was checked by FastQC (http://www.bioinformatics.babraham.ac.uk/projects/fastqc). Read parts with quality score less than 26 were trimmed and reads shorter than 50bp were discarded. F = forward sequence, R = reverse sequence. Table S2. Length of sequenced RNA reads. Four RNA replicates selected from five samples at each pollination condition were sequenced with NextSeq500 platform (Illumina) applying paired-end sequencing (2×75 bp). Sequence quality was checked by FastQC (http://www.bioinformatics.babraham.ac.uk/projects/fastqc). Read parts with quality score less than 26 were trimmed and reads shorter than 40bp were discarded. F=forward sequence, R = reverse sequence. Table S6. Number of up- and down-regulated genes. Genes up-regulated (FC > 2.0, padj < 0.1) and down-regulated (FC < − 2.0, padj < 0.1) were selected. [file 12864_2021_7503_MOESM1_ESM.pptx]

## Slide 1
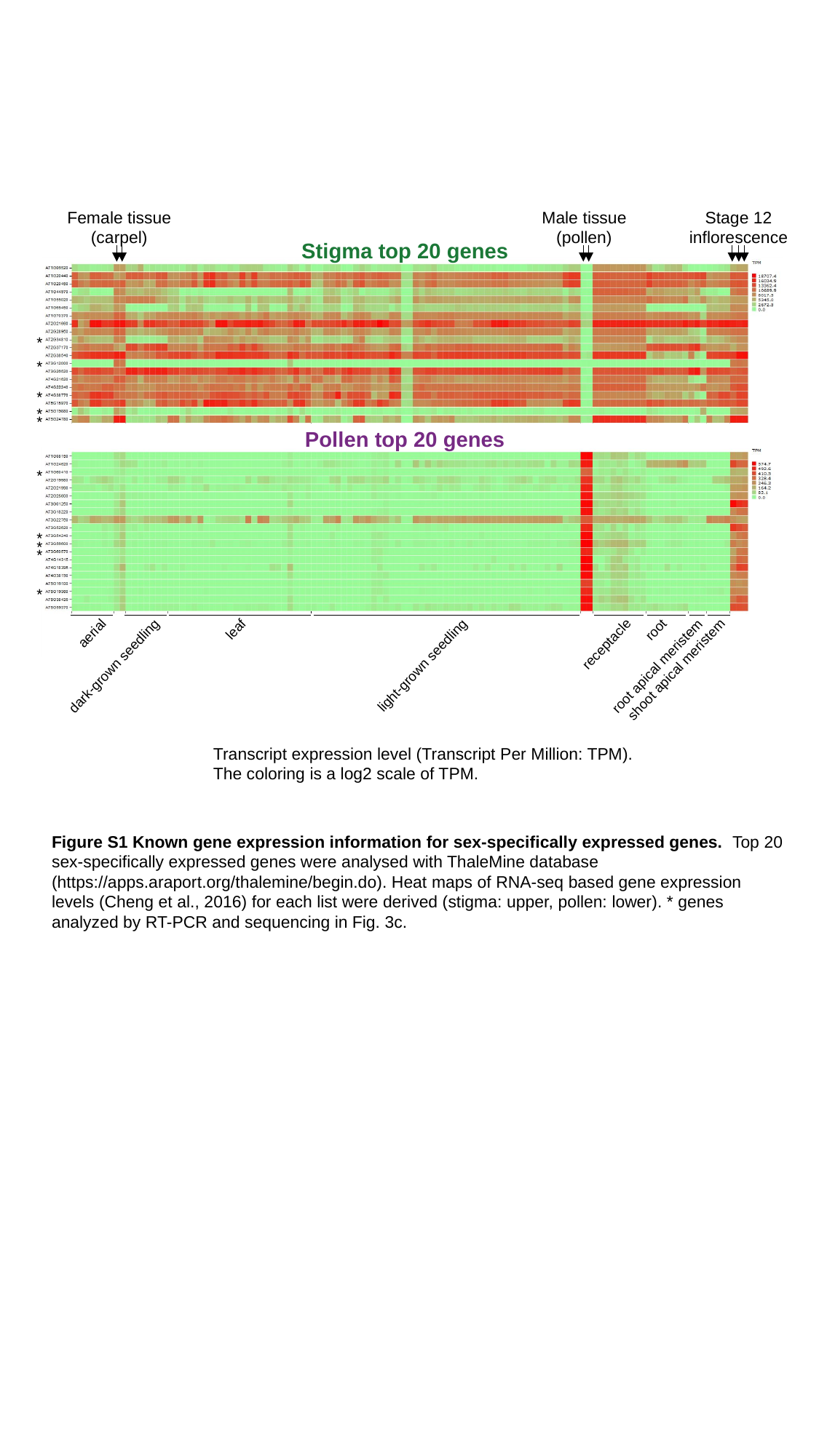

Female tissue
(carpel)
Male tissue
(pollen)
Stage 12 inflorescence
Stigma top 20 genes
*
*
*
*
*
Pollen top 20 genes
*
*
*
*
*
leaf
root
aerial
receptacle
light-grown seedling
dark-grown seedling
root apical meristem
shoot apical meristem
Transcript expression level (Transcript Per Million: TPM).
The coloring is a log2 scale of TPM.
Figure S1 Known gene expression information for sex-specifically expressed genes. Top 20 sex-specifically expressed genes were analysed with ThaleMine database (https://apps.araport.org/thalemine/begin.do). Heat maps of RNA-seq based gene expression levels (Cheng et al., 2016) for each list were derived (stigma: upper, pollen: lower). * genes analyzed by RT-PCR and sequencing in Fig. 3c.

## Slide 2
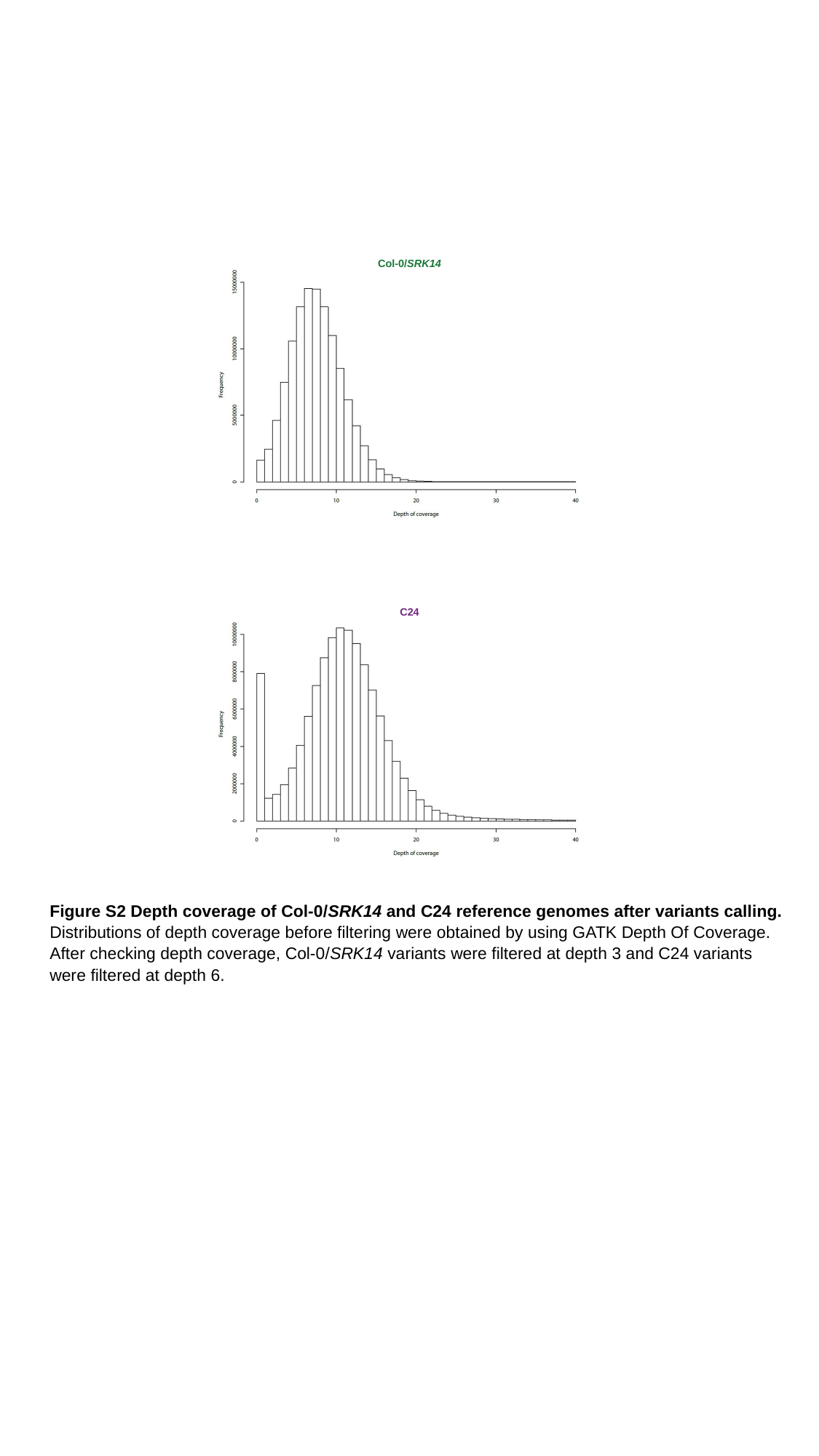

Col-0/SRK14
C24
Figure S2 Depth coverage of Col-0/SRK14 and C24 reference genomes after variants calling. Distributions of depth coverage before filtering were obtained by using GATK Depth Of Coverage. After checking depth coverage, Col-0/SRK14 variants were filtered at depth 3 and C24 variants were filtered at depth 6.

## Slide 3
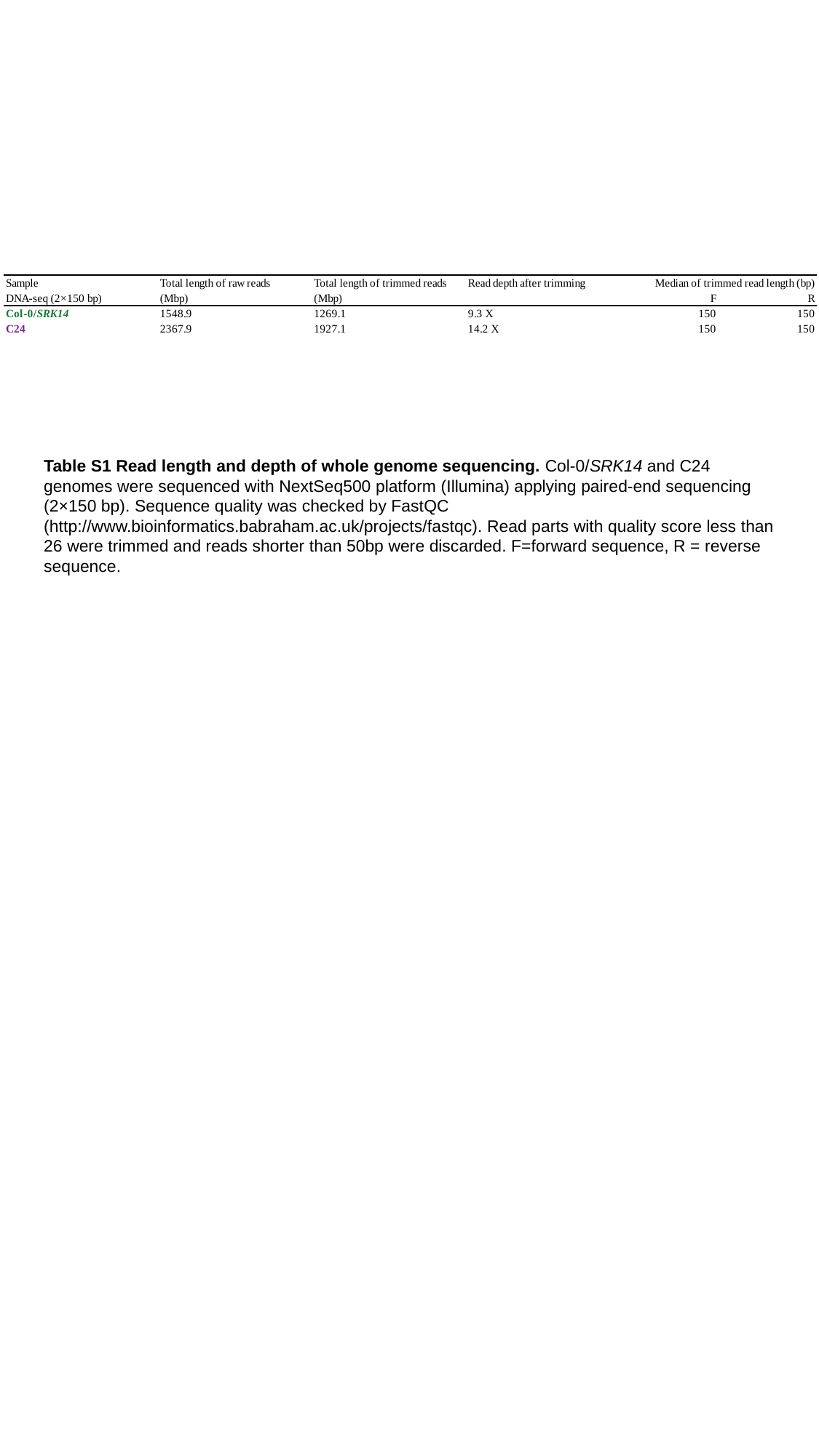

Table S1 Read length and depth of whole genome sequencing. Col-0/SRK14 and C24 genomes were sequenced with NextSeq500 platform (Illumina) applying paired-end sequencing (2×150 bp). Sequence quality was checked by FastQC (http://www.bioinformatics.babraham.ac.uk/projects/fastqc). Read parts with quality score less than 26 were trimmed and reads shorter than 50bp were discarded. F=forward sequence, R = reverse sequence.

## Slide 4
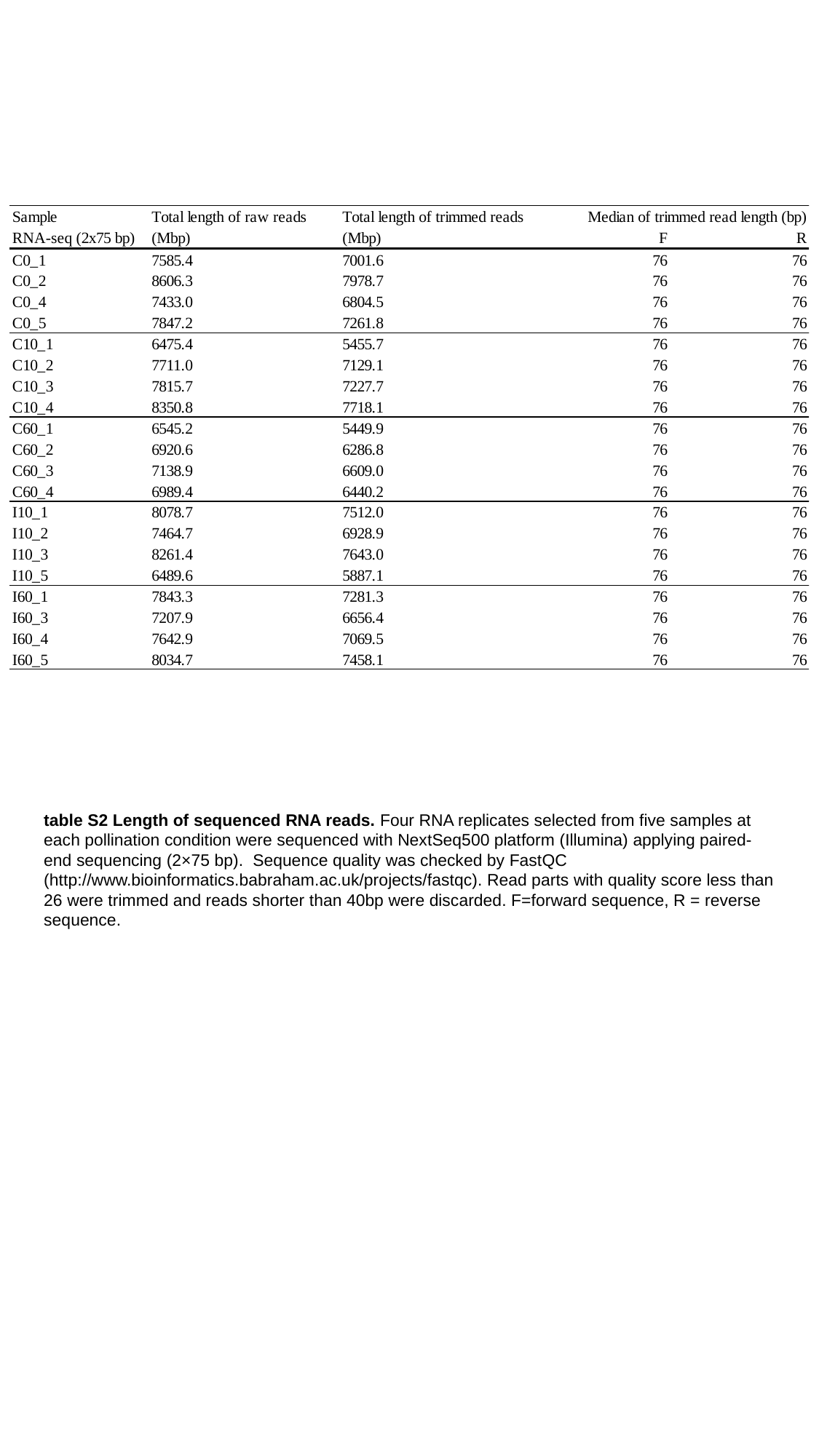

table S2 Length of sequenced RNA reads. Four RNA replicates selected from five samples at each pollination condition were sequenced with NextSeq500 platform (Illumina) applying paired-end sequencing (2×75 bp). Sequence quality was checked by FastQC (http://www.bioinformatics.babraham.ac.uk/projects/fastqc). Read parts with quality score less than 26 were trimmed and reads shorter than 40bp were discarded. F=forward sequence, R = reverse sequence.

## Slide 5
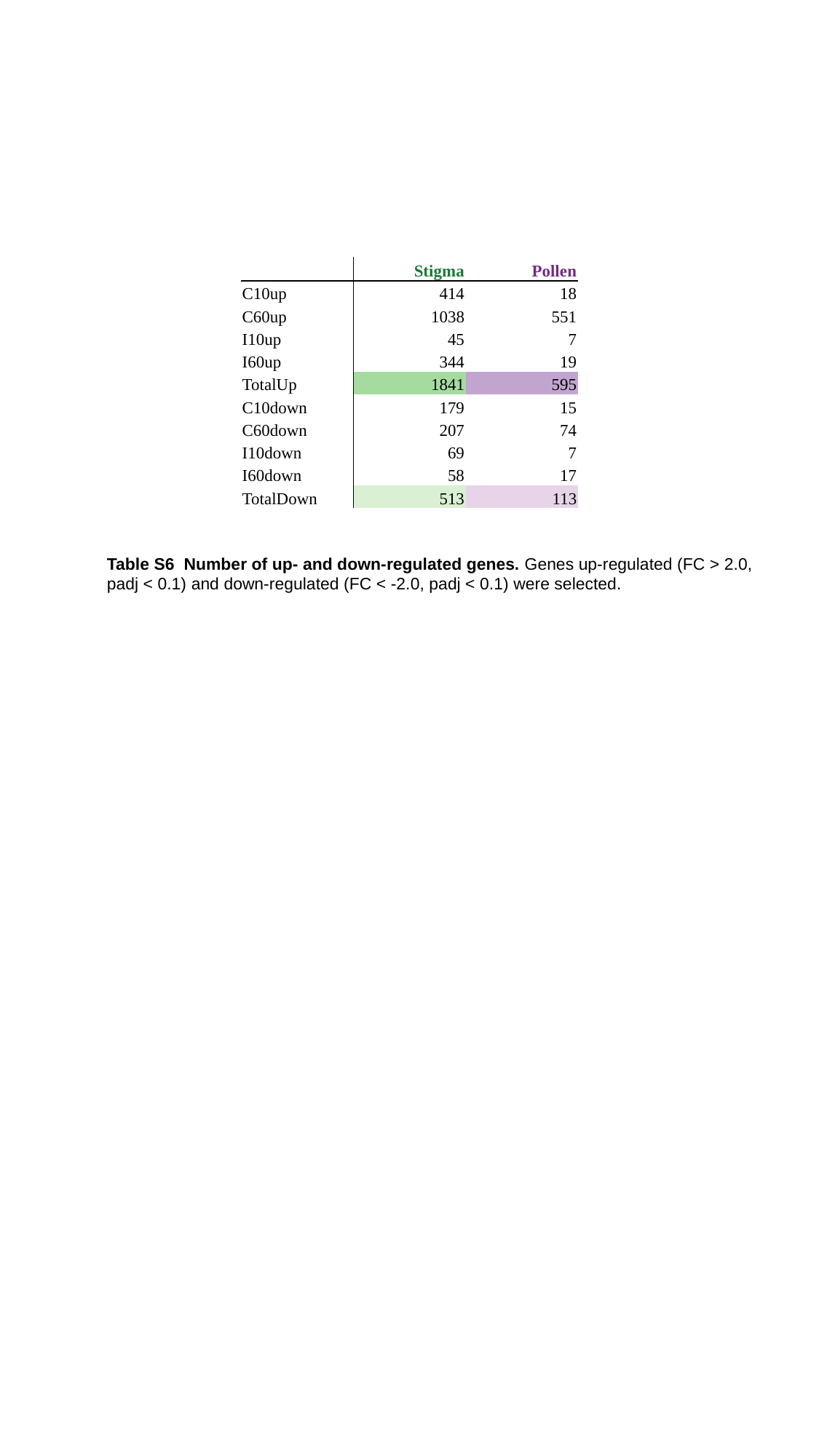

| | Stigma | Pollen |
| --- | --- | --- |
| C10up | 414 | 18 |
| C60up | 1038 | 551 |
| I10up | 45 | 7 |
| I60up | 344 | 19 |
| TotalUp | 1841 | 595 |
| C10down | 179 | 15 |
| C60down | 207 | 74 |
| I10down | 69 | 7 |
| I60down | 58 | 17 |
| TotalDown | 513 | 113 |
Table S6 Number of up- and down-regulated genes. Genes up-regulated (FC > 2.0, padj < 0.1) and down-regulated (FC < -2.0, padj < 0.1) were selected.
